# Systematic oxidative stress indices predicts prognosis in patients with urothelial carcinoma of the upper urinary tract after radical nephroureterectomy

**DOI:** 10.1186/s40001-023-01295-0

**Published:** 2023-10-28

**Authors:** Jianyong Liu, Shicong Lai, Pengjie Wu, Jiawen Wang, Jianye Wang, Jianlong Wang, Yaoguang Zhang

**Affiliations:** 1 Department of Urology, Beijing Hospital, National Center of Gerontology, Institute of the Geriatric Medicine, Chinese Academy of Medical Sciences, No. 1 DaHua Road, Dong Dan, Beijing, 100730 People’s Republic of China; 2 Graduate School of Peking Union Medical College and Chinese Academy of Medical Sciences, Beijing, People’s Republic of China; 3 Beijing Hospital Continence Center, Beijing, People’s Republic of China; 4 Department of Urology, Peking University People’s Hospital, Beijing, 100044 People’s Republic of China

**Keywords:** Upper urinary tract, Urothelial carcinoma, Systematic oxidative stress indices (SOSIs), Prognosis, Radical nephroureterectomy

## Abstract

**Background:**

Oxidative stress plays an important role in the occurrence and development of malignancy. However, the relationship between oxidative stress and upper urinary tract urothelial carcinoma (UTUC) prognosis remains elusive. This study aimed to evaluate the prognostic value of systematic oxidative stress indices as a predictor of patient outcomes in UTUC after radical nephroureterectomy.

**Methods:**

Clinical data for 483 patients with UTUC who underwent radical nephroureterectomy were analyzed. Patients were categorized according to an optimal value of systematic oxidative stress indices (SOSIs), including fibrinogen (Fib), gamma-glutamyl transpeptidase (γ-GGT), creatinine (CRE), lactate dehydrogenase (LDH) and albumin (ALB). Kaplan–Meier analyses were used to investigate associations of SOSIs with overall survival (OS) and progression-free survival (PFS). Moreover, associations between SOSIs and OS and PFS were assessed with univariate and multivariate analyses.

**Results:**

High values of Fib, γ-GGT, CRE, and LDH, and low values of ALB were associated with reduced OS. SOSIs status correlated with age, tumor site, surgical approach, hydronephrosis, tumor size, T stage, and lymph node status. The Kaplan–Meier survival analysis showed a significant discriminatory ability for death and progression risks in the two groups based on SOSIs. Multivariate Cox proportional hazards models showed that SOSIs were an independent prognostic indicator for OS (*p* = 0.007) and PFS (*p* = 0.021). SOSIs and clinical variables were selected to establish a nomogram for OS. The 1-, 3-, and 5-year AUC values were 0.77, 0.78, and 0.81, respectively. Calibration curves of the nomogram showed high consistencies between the predicted and observed survival probability. Decision curve analysis curves showed that the nomogram could well predict the 1‐year, 3-year, and 5‐year OS.

**Conclusions:**

SOSIs are an independent unfavorable predictor of OS and PFS in patients diagnosed with UTUC undergoing RNU. Therefore, incorporating SOSIs into currently available clinical parameters may improve clinical decision-making.

## Introduction

Upper urinary tract urothelial carcinomas (UTUCs), which are derived from the urothelium along the urinary tract, are located in the upper (pyelocaliceal cavities and ureter) urinary tract. UTUCs account for only 5–10% of all urothelial carcinomas (UCs) [1]. Radical nephroureterectomy (RNU) with bladder cuff excision remains the gold standard treatment for localized upper urinary tract urothelial carcinoma (UTUC). Because 60% of UTUCs are invasive at diagnosis, they usually have very poor prognosis [1, 2]. The 5-year specific survival is < 50% for pT2/pT3 and < 10% for pT4 [3–5]. European Association of Urology guidelines indicate that postoperative prognostic factors, such as lymph node involvement, tumor stage, and grade, are related to oncological outcomes. However, postoperative information might not contribute to making pretreatment assessments. Thus, identification of preoperatively available prognostic factors may enable physicians to determine preoperative treatment strategies concerning clinically valuable decisions in UTUC.

Oxidative stress is defined as a relative excess of reactive oxygen species (ROS) compared with antioxidants. ROS have been linked to a whole range of diseases, including cardiovascular disease, neurodegenerative disorders, autoimmune disease, and many cancers. Previous studies have revealed that intricate crosstalk between myeloid cell-derived ROS, oxidative DNA damage, and tumor necrosis factor α-mediated signaling can initiate cancer and contribute to tumor promotion and progression [6]. In addition, ROS can damage DNA, leading to genetic lesions that initiate tumorigenicity and subsequent tumor progression [7]. TBARS (thiobarbituric acid reactive substances) represents a good indicator of oxidative stress. A retrospective analysis revealed that metastatic urothelial carcinoma patients with increased TBARS had worse prognosis [8]. In addition, Chang et al. indicated that underexpression of glutathione peroxidase 2 (GPX2), a gene associated with oxidative stress, is a significant independent prognostic factor of urothelial carcinoma [9]. These findings provide evidence that oxidative stress is closely related to urothelial carcinoma.

In recent years, considerable evidence has demonstrated that several plasma biomarkers are associated with increased oxidative stress [10–12]. Higher levels of serum albumin (ALB) and lactate dehydrogenase (LDH) were observed in patients with trauma compared with the Antiox group. Decreasing the OS level with antioxidant substances correlated significantly with better prognosis and outcome [12]. Creatinine (CRE) and blood urea nitrogen (BUN) were significantly increased in a sleep-deprived mouse model associated with increased oxidative stress [11]. The serum-level of gamma-glutamyl transferase (*γ*-GGT) was found to decrease after antioxidant therapy [13]. Some previous results demonstrate that fibrinogen during acute inflammatory states may be affected by oxidative stress and can be used as a marker to reflect the status of systematic oxidative stress [14–16]. Systematic oxidative stress has been proven to be useful as a predictor of prognosis in many cancers, including breast cancer, colorectal cancer, and T lymphoblastic lymphoma/leukemia [17–19]. However, the feasibility of oxidative stress for UTUC has not been evaluated to date.

Therefore, this study aimed to explore correlations of systematic oxidative stress indices (SOSIs) with clinicopathologic parameters and to validate the prognostic value of SOSIs as a predictor of patient outcome in UTUC after radical nephroureterectomy.

## Materials and methods

### Study population

This retrospective analysis evaluated data from patients without evidence of distant metastases who underwent RNU between March 1996 and June 2021 at Beijing Hospital, National Center of Gerontology, Institute of the Geriatric Medicine, Chinese Academy of Medical Sciences, Beijing. Patients who had not undergone RNU, had evidence of metastatic disease at the time of surgery, or had incomplete preoperative medical information on hematologic indicators; patients who were lost to follow-up; patients with autoimmune diseases; and patients who had received preoperative adjuvant chemotherapy, radiotherapy, or any other antitumor therapy were excluded from the study. Finally, we assessed data for a final total of 483 patients who underwent open or laparoscopic RNU.

### Data collection and evaluation

Data regarding sex, age, symptoms, tumor location, tumor side, tumor size, presence of preoperative hydronephrosis, multifocality, pathologic T and N stage, appearance of lymphovascular invasion, chemotherapy, tumor grade, positive surgical margin, surgical approach, and the presence of concomitant carcinoma in situ (CIS) were obtained from the Beijing Hospital Information System. Biochemical information, including fibrinogen (Fib), gamma-glutamyl transpeptidase (*γ*-GGT), creatinine (CRE), lactate dehydrogenase (LDH), albumin (ALB), blood urea nitrogen (BUN), and alkaline phosphatase (ALP), was obtained within 7 days before RNU. Progression-free survival (PFS), and overall survival (OS) were obtained from medical records. Surgical specimens were processed by an experienced pathologist, who confirmed the T stage (based on the American Joint Committee on Cancer TNM Classification, 7th edition), tumor grade (based on 1998 WHO classification), lympho-vascular invasion (LVI), and presence of variant histology.

### Follow-up regimen

Postoperative follow-up included routine urine tests, urine pathology chest radiography, computed tomography, and cystoscopy. Patients were generally assessed postoperatively every 3–4 months in the first year after RNU, every 6 months from the second year to the fifth year, and annually thereafter. PFS (measured from RNU until the date of last follow-up or date of disease progression (including local recurrence or distant metastasis or death) and OS (measured from RNU until the date of last follow-up or date of death from any cause) were selected as primary endpoints.

### Statistical analysis

Comparisons between the clinicopathological characteristics of the patients were performed using the chi-square test and Mann–Whitney *U* test, as appropriate. Seven plasma biomarkers associated with increased oxidative stress, including Fib, CRE, GGT, ALB, LDH, ALP, and BUN, were identified for calculating SOSIs, which were determined by the lowest Akaike information criterion (AIC) value. The value was defined as 1 if the plasma biomarker value was above the cutoff level; the value was defined as 0 if the plasma biomarkers value was below the cutoff level. Survival curves were generated using the Kaplan–Meier method with the log-rank test. Univariate and multivariate Cox proportional hazard models were conducted to evaluate the impact of variables on OS and PFS after RNU. Time-dependent receiver operating characteristic (ROC) analysis and decision curve analysis (DCA) were used to assess the prognostic capacity of the nomogram. A* P* value lower than 0.05 was considered statistically significant. Data analysis was completed with R software, version 4.2.1.

## Results

### Basic information of selected patients

The clinical and pathologic characteristics of the patients are shown in Table 1. In this cohort, males accounted for 51.1% (247 patients) and females for 48.9% (236 patients), with a median age of 70 years (interquartile range: 62–76). The cutoff values of Fib, CRE, GGT, ALB, BUN, ALP, and LDH derived from the ROC curves were 3.978 g/L, 83.5 μmol/L, 43.5 U/L, 39.5 g/L,9.07 mg/dL, 83.5 UL/L, and 170.5 U/L, respectively. During the median follow-up of 36.8 (interquartile range [IQR]: 22.0–68.5) months, a total of 182 (37.7%) patients died, and 224 (46.4%) had progressive disease.

**Table 1:** Clinical characteristics of the study population

Characteristics	Number (percentage)
Sex	
Female	247 (51.1%)
Male	236 (48.9%)
Age	
< 65	150 (31.1%)
≥ 65	333 (68.9%)
BMI	
< 25	287 (59.4%)
≥ 25	196 (40.6%)
Side	
Left	273 (56.5%)
Right	210 (43.5%)
Site	
Both	31 (6.42%)
Pelvis	206 (42.7%)
Ureter	246 (50.9%)
Approach	
Laparoscopic	282 (58.4%)
Open	201 (41.6%)
Ureteroscopy	
No	389 (80.5%)
Yes	94 (19.5%)
Hematuresis	
No	128 (26.5%)
Yes	355 (73.5%)
Urine pathology	
No	132 (27.3%)
Yes	351 (72.7%)
Hydronephrosis	
No	110 (22.8%)
Yes	373 (77.2%)
Multifocality	
No	418 (86.5%)
Yes	65 (13.5%)
Size	
< 5	416 (86.1%)
≥ 5	67 (13.9%)
LVI	
No	413 (85.5%)
Yes	70 (14.5%)
Tis	
No	460 (95.2%)
Yes	23 (4.76%)
T stage	
T1	128 (26.5%)
T2	143 (29.6%)
T3	190 (39.3%)
T4	22 (4.55%)
Margin	
Negative	470 (97.3%)
Positive	13 (2.69%)
pN +	
N0&Nx	450 (93.2%)
N +	33 (6.83%)
Grade	
High	389 (80.5%)
Low	94 (19.5%)

*BMI* body mass index, *CIS* carcinoma in situ, *LVI* lymphovascular invasion

### Calculation of systematic oxidative stress indices (SOSIs) and baseline characteristics of UTUC patients in different SOSI groups

**Fig. 1:**
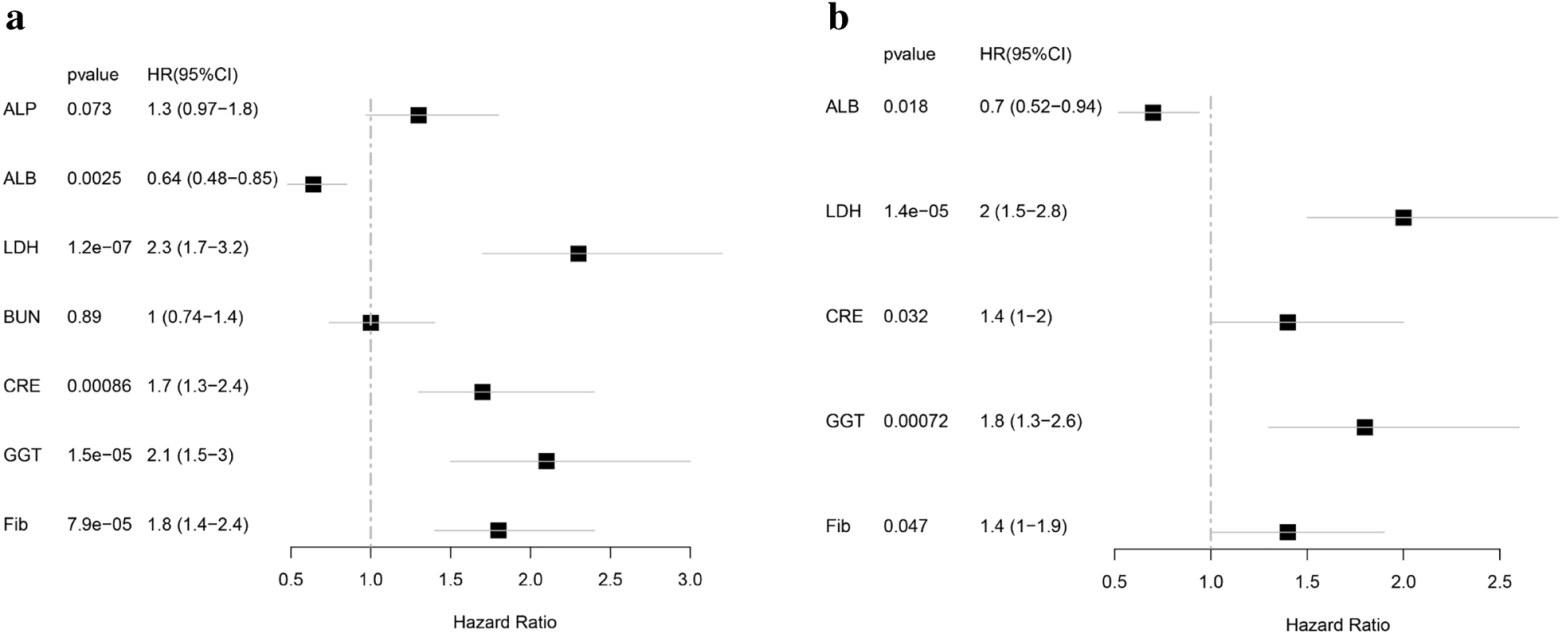
SOSI is related to OS in upper urinary tract urothelial carcinoma patients. **a**, **b** systematic oxidative stress indices were included in univariate and multivariate Cox regression analyses

First, we divided the seven plasma biomarkers into 2 groups using the cutoff value. Values of “0” and “1” were used for scoring according to the algorithm mentioned above. Univariate and multivariate Cox regression analyses were used to explore whether the above seven plasma biomarkers are independent prognostic factors for UTUC. According to univariate Cox regression analysis, we found that Fib, GGT, CRE, BUN, LDH, and ALB correlated significantly with overall survival (OS) (all *p* < 0.05) (Fig. 1a). Multivariate Cox regression analysis indicated that Fib, GGT, CRE, LDH, and ALB were associated with OS (Fig. 1b). Then, the SOSIs prognostic model was generated based on the lowest AIC value: SOSIs = Fib*0.3141 + GGT*0.6059 + CRE*0.3582 + LDH*0.7149–ALB*0.3600. The cutoff value of SOSIs based on the median value was set as 0.3582 and used to divide the patients into high-risk (272 with SOSIs of 0.3582 or above) and low-risk groups (211 with a SOSIs level less 0.3582). The clinical characteristics of all the patients grouped by SOSIs are presented in Table 2. Patients in the high-risk group had higher incidences of ≥ pT3 stage and pN + . In the high-risk patient group, tumor size, hydronephrosis, and positive urine pathology were significantly higher than those in the low-risk patient group. There was an obvious difference between the two groups in terms of age, tumor site, and surgical approach. However, no significant difference was found for sex, BMI, tumor side, previous ureteroscopic surgical margin status, multifocality, presence of lymphovascular invasion, chemotherapy, lymph node stage, presence of CIS in surgical specimens, or tumor grade.

**Table 2:** Baseline and clinicopathological characteristics of UTUC patients

	High Risk	Low Risk	*p*.overall
	N = 272	N = 211	
Sex			0.769
Female	137 (50.4%)	110 (52.1%)	
Male	135 (49.6%)	101 (47.9%)	
Age			< 0.001
< 65	59 (21.7%)	91 (43.1%)	
≥ 65	213 (78.3%)	120 (56.9%)	
BMI			0.253
< 25	155 (57.0%)	132 (62.6%)	
≥ 25	117 (43.0%)	79 (37.4%)	
Side			0.211
Left	161 (59.2%)	112 (53.1%)	
Right	111 (40.8%)	99 (46.9%)	
Site			0.001
Both	24 (8.82%)	7 (3.32%)	
Pelvis	99 (36.4%)	107 (50.7%)	
Ureter	149 (54.8%)	97 (46.0%)	
Approach			< 0.001
Laparoscopic	132 (48.5%)	150 (71.1%)	
Open	140 (51.5%)	61 (28.9%)	
Ureteroscopy			0.085
No	227 (83.5%)	162 (76.8%)	
Yes	45 (16.5%)	49 (23.2%)	
Hematuresis			0.904
No	71 (26.1%)	57 (27.0%)	
Yes	201 (73.9%)	154 (73.0%)	
Urine pathology			0.015
No	62 (22.8%)	70 (33.2%)	
Yes	210 (77.2%)	141 (66.8%)	
Hydronephrosis			< 0.001
No	43 (15.8%)	67 (31.8%)	
Yes	229 (84.2%)	144 (68.2%)	
Multifocality			0.064
No	228 (83.8%)	190 (90.0%)	
Yes	44 (16.2%)	21 (9.95%)	
Size			0.020
< 5	225 (82.7%)	191 (90.5%)	
≥ 5	47 (17.3%)	20 (9.48%)	
LVI			0.288
No	228 (83.8%)	185 (87.7%)	
Yes	44 (16.2%)	26 (12.3%)	
Tis			0.505
No	257 (94.5%)	203 (96.2%)	
Yes	15 (5.51%)	8 (3.79%)	
T stage			0.002
T1	59 (21.7%)	69 (32.7%)	
T2	86 (31.6%)	57 (27.0%)	
T3	108 (39.7%)	82 (38.9%)	
T4	19 (6.99%)	3 (1.42%)	
Margin			0.072
Negative	261 (96.0%)	209 (99.1%)	
Positive	11 (4.04%)	2 (0.95%)	
pN +			0.012
N0&Nx	246 (90.4%)	204 (96.7%)	
N +	26 (9.56%)	7 (3.32%)	
Chemotherapy			0.987
No	223 (82%)	174 (82,5%)	
Yes	49 (18%)	37 (17.5%)	
Grade			1.000
High	219 (80.5%)	170 (80.6%)	
Low	53 (19.5%)	41 (19.4%)	

*BMI* body mass index, *CIS* carcinoma in situ, *LVI* lymphovascular invasion

### Relationship between SOSIs and clinical features

**Fig. 2:**
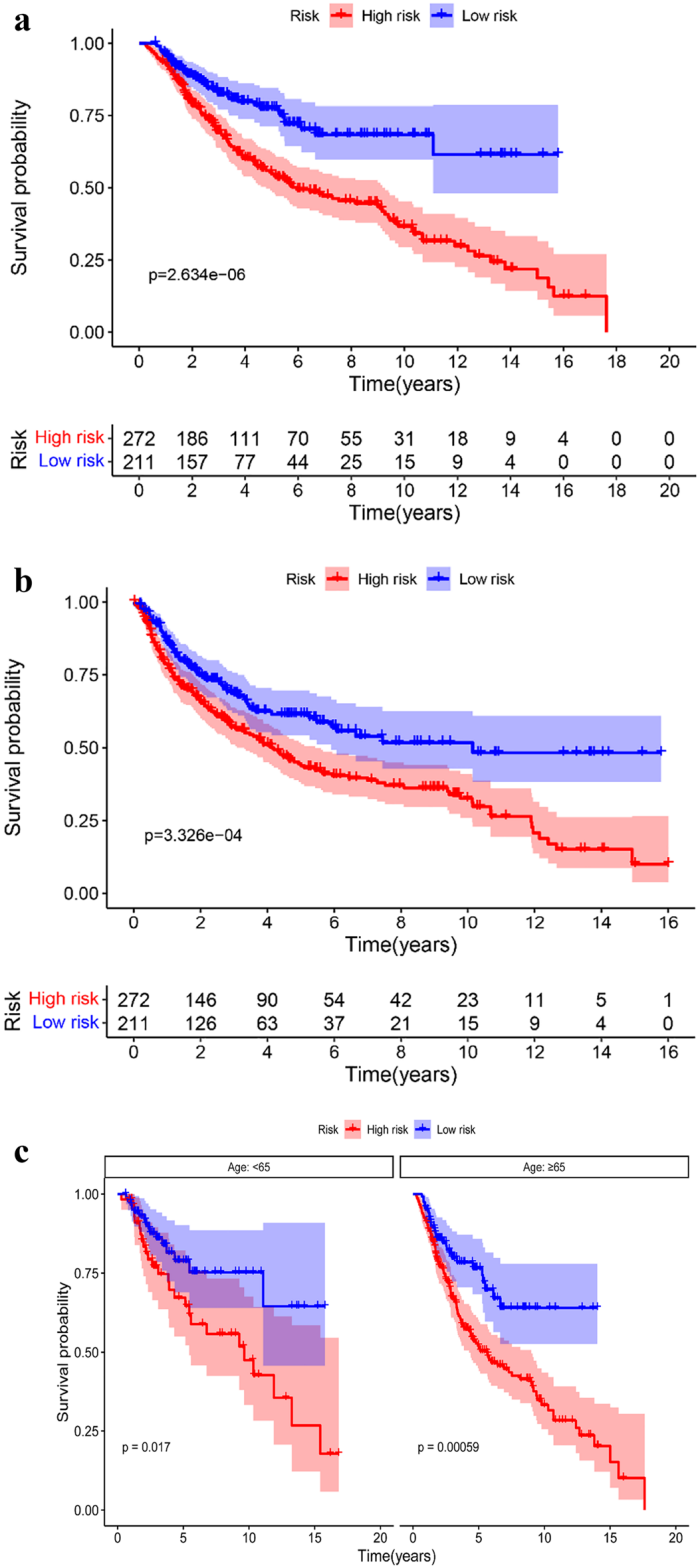

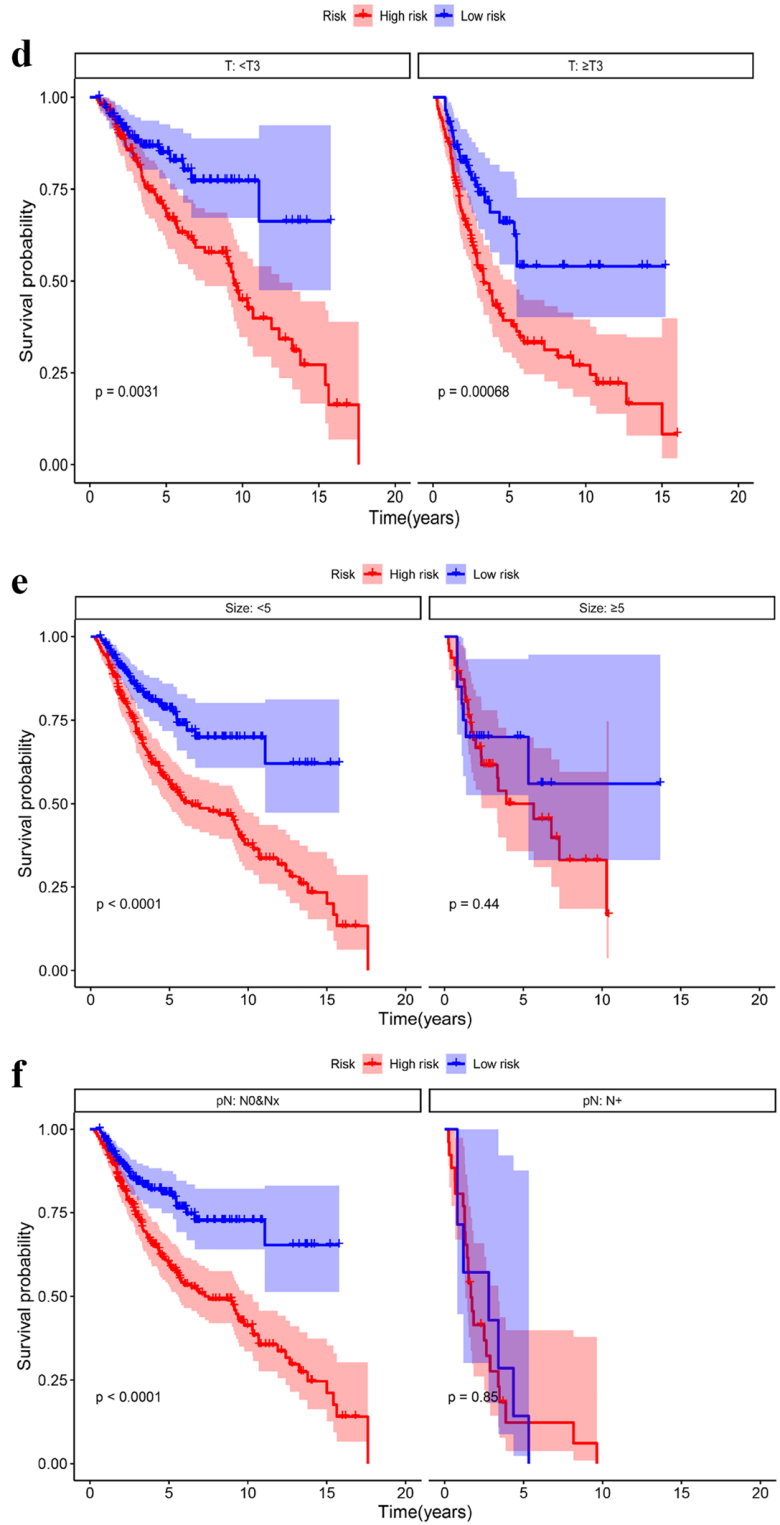

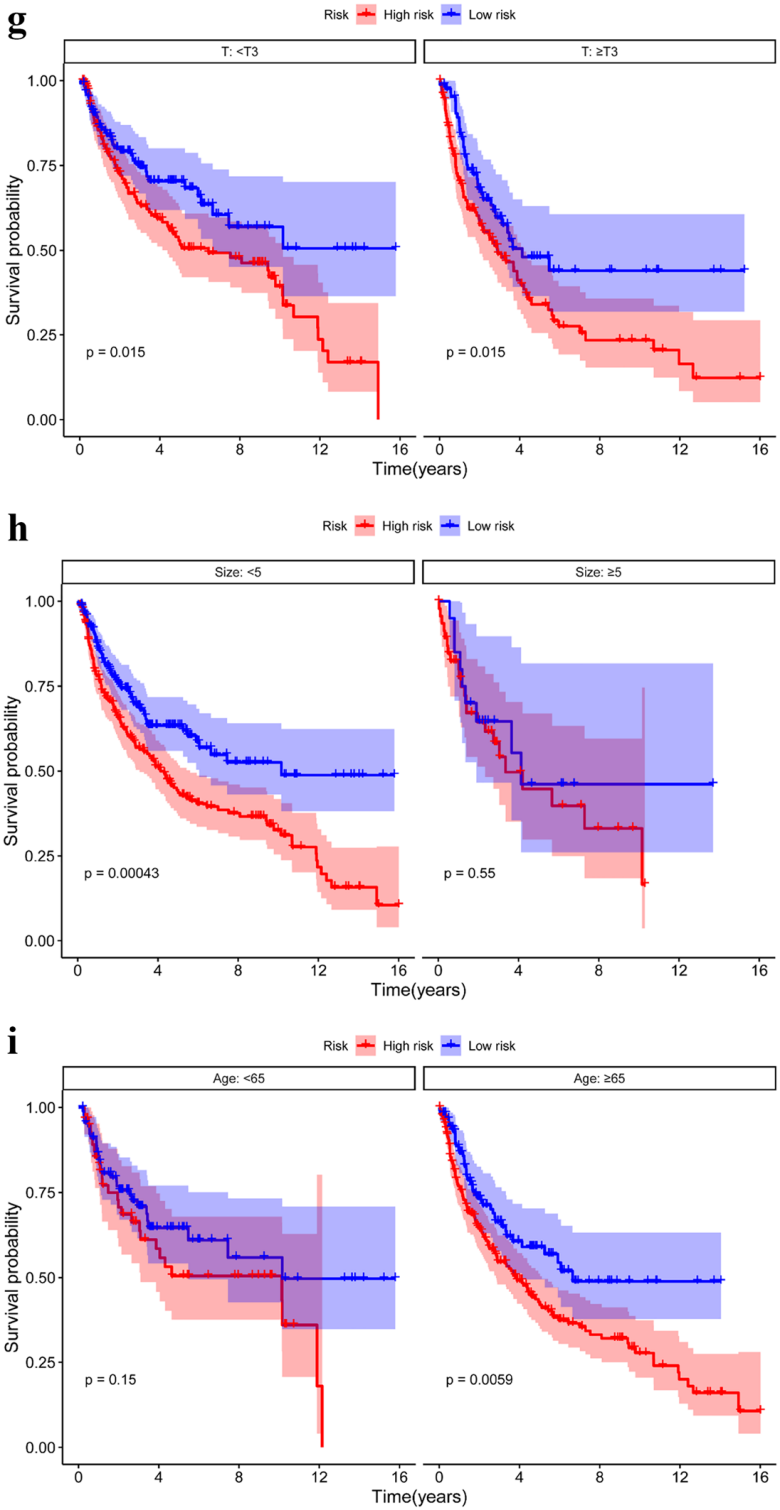

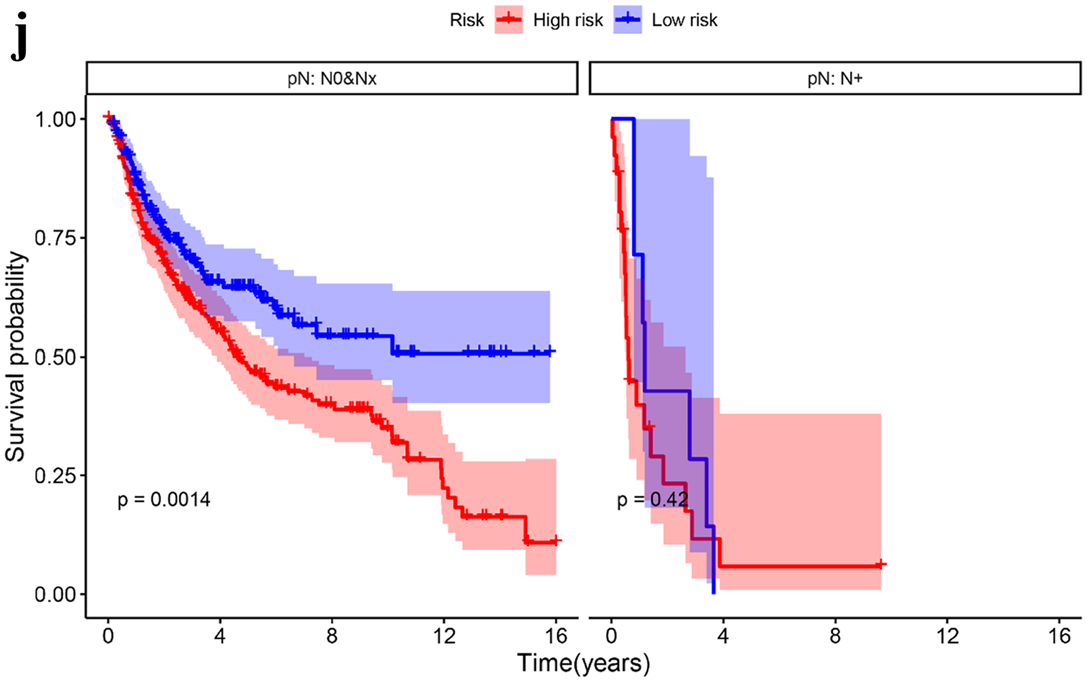
Kaplan–Meier curves for OS and PFS based on SOSIs. OS (**a**) and PFS (**b**) in the cohort; Subgroup analysis based on age (**c**), T stage (**d**), size (**e**) and pN (**f**), Kaplan–Meier curves for OS stratified according to SOSIs for UTUC patients receiving RNU; Subgroup analysis based on T stage (**g**), size (**h**), age (**i**) and pN (**j**), Kaplan–Meier curves for PFS stratified according to SOSIs for UTUC patients receiving RNU

The Kaplan–Meier curve showed that the patients in the high-risk group had worse overall survival (OS) (Fig. 2a) and progression-free survival (PFS) (Fig. 2b) than those in the low-risk group. We also found that OS was worse in the high-risk group than in the low-risk group in subgroup analysis performed by age (Fig. 2c) and T stage (Fig. 2d). In the size ≤ 5 and pN0&Nx groups, the high-risk group had worse prognosis, but there was no significant difference in the size > 5 and pN + groups between the two groups (Fig. 2e, f). Subgroup analyses suggested a significant difference in PFS between the two risk groups for the two T-stage groups (Fig. 2g). In size ≤ 5 (Fig. 2h), age ≥ 65 (Fig. 2i) and pN0&Nx (Fig. 2j) subgroups, survival analysis showed that patients with low-risk had significantly favorable PFS compared with patients with high-risk.

### Evaluation of SOSIs as an independent prognostic factor in patients with UTUC

Univariate and multivariate analyses were performed to determine predictors of OS (Table 3). In univariate analyses,we found that age (HR = 1.681,* p* = 0.003), tumor site at the ureter (HR = 1.413,* p* = 0.029), use of ureteroscopy (HR = 1.620,* p* = 0.014), hydronephrosis (HR = 1.904,* p* = 0.001), size ≥ 5 (HR = 1.744,* p* = 0.005), LVI (HR = 3.766,* p* < 0.001), T stage (T2, HR = 2.507, *p* < 0.001; T3, HR = 3.404, *p* < 0.001; T4, HR = 12.446, *p* < 0.001), margin (HR = 5.062,* p* < 0.001), N stage (HR = 5.344,* p* < 0.001), grade (HR = 2.013,* p* = 0.001), chemotherapy (HR = 2.241,* p* < 0.001), and SOSIs (HR = 2.217,* p* < 0.001) were significantly associated with OS. Those significant and potential risk factors were evaluated in multivariate analyses, in which OS (all *p* < 0.05)was independently predicted by age, LVI, T stage, N stage, margin, and SOSIs.

**Table 3:** Univariate and multivariate Cox analyses for OS of UTUC patients

Variable	Univariate Cox analysis			Multivariate Cox analysis		
	HR	95%CI	*p* value	HR	95%CI	*p* value
Sex						
Female	Reference					
Male	1.257	0.938–1.683	0.125			
Age						
< 65	Reference			Reference		
≥ 65	1.681	1.189–2.376	0.003	1.509	1.036–2.196	0.032
BMI						
< 25	Reference					
≥ 25	0.936	0.695–1.260	0.663			
Side						
Left	Reference					
Right	1.019	0.760–1.367	0.899			
Site						
Pelvis	Reference			Reference		
Ureter	1.413	1.036–1.928	0.029	1.244	0.860–1.801	0.246
Both	1.037	0.571–1.883	0.905	0.685	0.387–1.328	0.232
Approach						
Open	Reference					
Laparoscopic	1.132	0.836–1.532	0.423			
Ureteroscopy						
No	Reference			Reference		
Yes	1.620	1.101–2.384	0.014	1.171	0.752–1.824	0.485
Hydronephrosis						
No	Reference			Reference		
Yes	1.904	1.285–2.821	0.001	1.217	0.781–1.897	0.385
Multifocality						
No	Reference					
Yes	1.318	0.909–1.911	0.145			
Size						
< 5	Reference			Reference		
≥ 5	1.744	1.181–2.574	0.005	1.205	0.776–1.872	0.407
LVI						
No	Reference			Reference		
Yes	3.766	2.668–5.316	< 0.001	1.979	1.276–3.069	0.002
Tis						
No	Reference					
Yes	1.018	0.520–1.992	0.958			
T stage						
T1	Reference			Reference		
T2	2.507	1.551–4.052	< 0.001	1.804	1.094–2.974	0.021
T3	3.404	2.155–5.376	< 0.001	2.334	1.408–3.870	0.001
T4	12.446	6.821–22.709	< 0.001	3.633	1.647–8.014	0.001
Margin						
Negative	Reference			Reference		
Positive	5.062	2.648–9.678	< 0.001	2.999	1.497–6.006	0.002
N stage						
pN0&Nx	Reference			Reference		
pN +	5.344	3.583–7.972	< 0.001	2.191	1.342–3.576	0.002
Grade						
Low						
High	2.013	1.354–2.993	0.001	1.357	0.884–2.084	0.163
Chemotherapy						
No	Reference			Reference		
Yes	2.241	1.611–3.116	< 0.001	1.274	0.869–1.868	0.214
SOSI						
Low risk	Reference			Reference		
High risk	2.217	1.577–3.117	< 0.001	1.656	1.147–2.392	0.007

*BMI* body mass index, *CIS* carcinoma in situ, *LVI* lymphovascular invasion

Interestingly, sex (HR = 1.335,* p* = 0.030), age (HR = 1.435,* p* = 0.018), tumor site at the ureter (HR = 1.617,* p* = 0.001), use of ureteroscopy (HR = 1.489,* p* = 0.025), hydronephrosis (HR = 1.544,* p* = 0.010), LVI (HR = 2.467,* p* < 0.001), T stage (T2, HR = 1.832, *p* = 0.002; T3, HR = 2.098, *p* < 0.001; T4, HR = 5.538, *p* < 0.001), margin (HR = 2.308,* p* = 0.021), N stage (HR = 4.302,* p* < 0.001), grade (HR = 1.809,* p* = 0.001), and SOSI (HR = 1.721,* p* < 0.001) were all highly significantly different in univariate PFS analysis. Next, age, tumor site at the ureter, LVI, T stage, N stage, and SOSIs were all significantly identified in PFS multivariate analysis (Table 4).

**Table 4:** Univariate and multivariate Cox analyses for PFS of UTUC patients

Variable	Univariate cox analysis			Multivariate cox analysis		
	HR	95%CI	p value	HR	95%CI	*p* value
Sex						
Female	Reference			Reference		
Male	1.335	1.029–1.732	0.030	1.220	0.928–1.640	0.154
Age						
< 65	Reference			Reference		
≥ 65	1.435	1.065–1.933	0.018	1.393	1.001–1.937	0.049
BMI						
< 25	Reference					
≥ 25	0.864	0.662–1.128	0.282			
Side						
Left	Reference					
Right	1.094	0.843–1.419	0.499			
Site						
Pelvis	Reference			Reference		
Ureter	1.617	1.228–2.130	0.001	1.520	1.107–2.089	0.010
Both	1.000	0.567–1.766	0.999	0.787	0.438–1.412	0.421
Approach						
Open	Reference					
Laparoscopic	1.163	0.892–1.517	0.265			
Ureteroscopy						
No	Reference			Reference		
Yes	1.489	1.051–2.111	0.025	1.250	0.856–1.827	0.248
Hydronephrosis						
No	Reference			Reference		
Yes	1.544	1.110–2.148	0.010	1.039	0.713–1.515	0.842
Multifocality						
No	Reference					
Yes	1.038	0.721–1.493	0.842			
Size						
< 5	Reference					
≥ 5	1.217	0.841–1.762	0.298			
LVI						
No	Reference			Reference		
Yes	2.467	1.764–3.451	< 0.001	1.708	1.142–2.554	0.009
Tis						
No	Reference					
Yes	0.927	0.492–1.748	0.815			
T stage						
T1	Reference			Reference		
T2	1.823	1.240–2.679	0.002	1.438	0.899–2.020	0.148
T3	2.098	1.457–3.021	< 0.001	1.610	1.079–2.403	0.020
T4	5.538	3.134–9.785	< 0.001	2.111	1.049–4.244	0.036
Margin						
Negative	Reference			Reference		
Positive	2.308	1.135–4.694	0.021	1.727	0.860–3.468	0.125
N stage						
pN0&Nx	Reference			Reference		
pN +	4.302	2.858–6.476	< 0.001	2.767	1.747–4.382	< 0.001
Grade						
Low						
High	1.809	1.280–2.556	0.001	1.432	0.982–2.061	0.062
Chemotherapy						
No	Reference					
Yes	1.177	0.841–1.647	0.342			
SOSI						
Low risk	Reference			Reference		
High risk	1.721	1.303–2.273	< 0.001	1.430	1.055–1.937	0.021

*BMI* body mass index, *CIS* carcinoma in situ, *LVI* lymphovascular invasion

### Development and validation of the nomogram prediction model

**Fig. 3:**
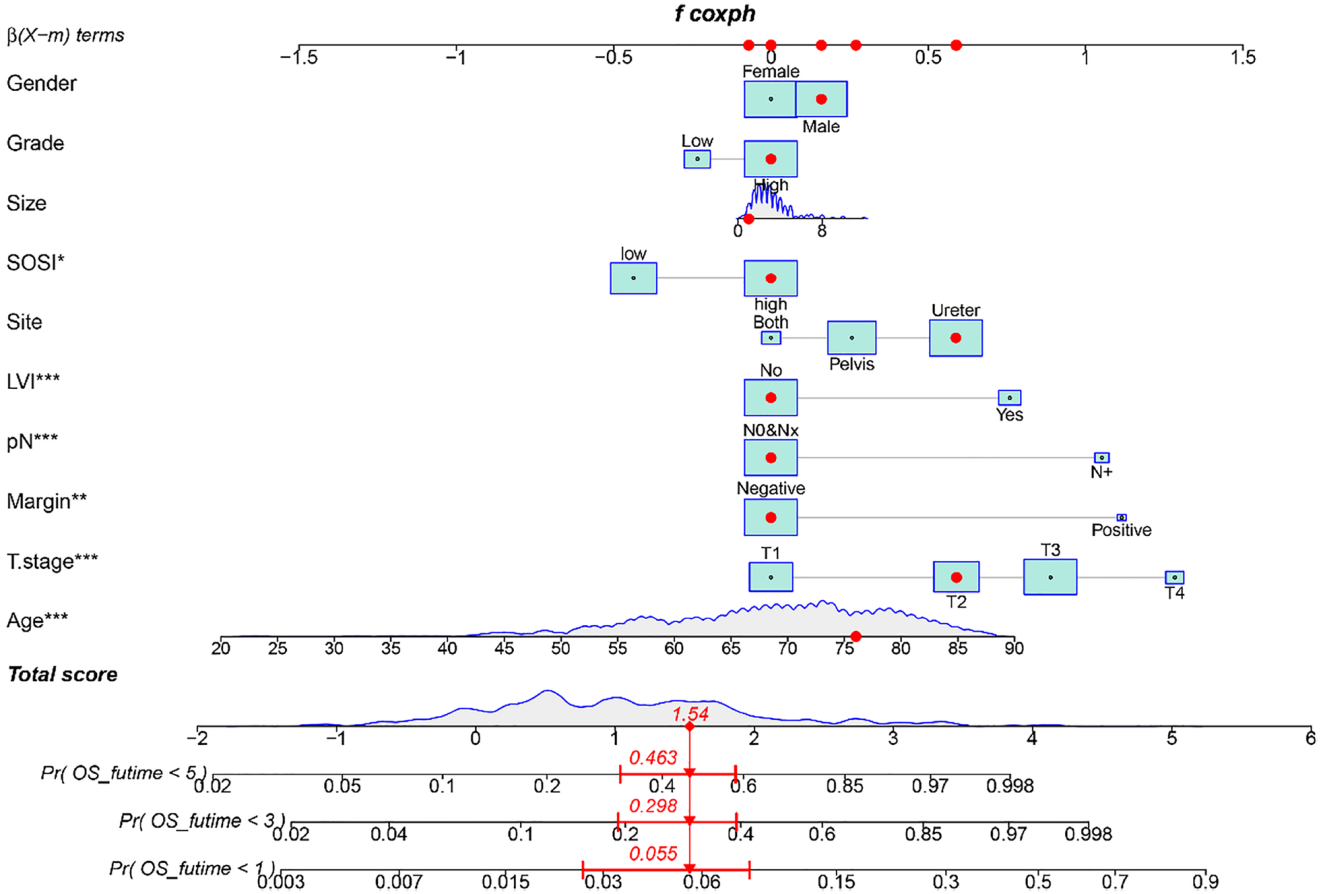
Nomogram constructed for prognostic prediction of upper urinary tract urothelial carcinoma

**Fig. 4:**
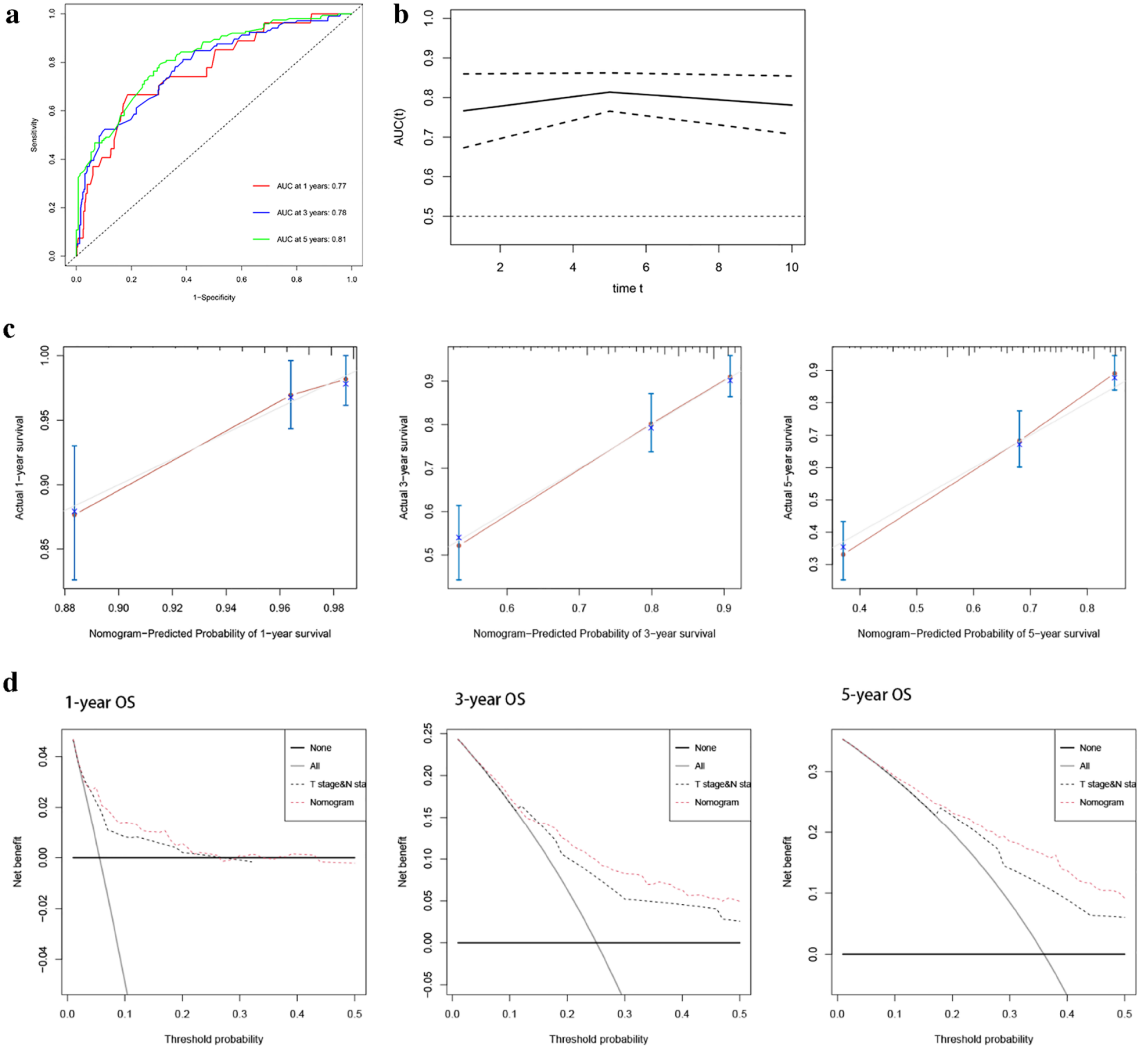
Predictive accuracy of the nomogram. **a**, **b** Time-dependent ROC curves were used to determine the prognostic value of the nomogram. **c** Calibration curves of 1‐year, 3‐year and 5‐year OS for UTUC patients in the cohort. **d** Decision curve analysis for OS in UTUC patients

The independent predictors were used to construct a nomogram (Fig. 3). The areas under the curve (AUCs) of the nomogram for predicting 1-year, 3-year, and 5-year survival rates were 0.77, 0.78, and 0.81, respectively (Fig. 4a). The time‐dependent AUC was > 0.7 for prediction of OS within 10 years, indicating favorable discrimination by the nomogram (Fig. 4b). As shown in Fig. 4c, calibration curves indicated good agreement between the predicted and observed probabilities. Furthermore, in decision curve analysis, the nomogram consistently achieved a greater net benefit than traditional prognostic indicators (Fig. 4d). Collectively, these results indicate that the nomogram has good concordance and accuracy.

## Discussion

In the present study, we investigated systematic oxidative stress indices levels in 483 patients undergoing RNU for localized UTUC. Elevated preoperative SOSIs levels were associated with higher incidences of ≥ pT3 stage and pN + . Kaplan–Meier analysis showed that patients with high SOSIs levels were predicted to have poor overall survival and progression-free survival. Furthermore, results from multivariate analysis confirmed SOSIs levels to be an independent predictor of OS and PFS. Using SOSIs and clinical–pathological features, we developed a nomogram to predict survival outcomes for patients with UTUC. To the best of our knowledge, this is the first report to describe an association of combined SOSIs with prognosis in upper urinary tract urothelial carcinoma after radical nephroureterectomy. The results suggest that SOSIs levels are a strong biomarker for predicting oncological outcomes preoperatively in localized UTUC.

The concept of oxidative stress was first proposed in 1985 for redox biology and medicine research, approximately 40 years ago [20]. Since then, redox biology as a field of study has exploded in a wide range of disciplines. Imbalance between oxidants (so-called reactive oxygen species, ROS) and antioxidants is the core of oxidative stress [21]. This imbalance leads to damage to important biomolecules and cells, with a potential impact on the whole organism [22]. In recent years, considerable evidence has demonstrated that ROS may increase the risk of cancer when the balance between the relative abundance of ROS and antioxidants is broken. Sod1-deficient mice develop liver cancer, as marked by extensive oxidative and DNA damage. Mice heterozygous for a null allele of Sod2 also exhibit increased levels of oxidative damage to DNA and form tumors, particularly lymphoma and pituitary adenoma [23–25]. Many studies have shown the significant role of oxidative stress in the development and progression of malignancies, such as breast cancer, hepatocellular carcinoma, and prostate cancer, through excessive production of reactive oxygen species [26–28]. As one of the key antioxidant enzymes, GPX2 plays an important role in catalyzing reduction of hydrogen peroxide or organic hydroperoxides [29]. Chang et al. found that underexpression of GPX2 in UTUC correlated strongly with more advanced and more aggressive disease. In addition, GPX2 underexpression and downregulation are predictive of poor prognosis in patients with UTUC [9].

Increasing evidence has consistently shown the role of SOSIs in predicting patient outcomes in multiple malignant tumors [17–19]. Our findings are consistent with those of previous studies, as SOSIs are considered an independent predictor. Fib is an adhesive plasma protein that plays a central role in hemostasis. Previous work has revealed that Fib is targeted for oxidative modifications in *vivo,* and that it can reflect the status of systematic oxidative stress [15, 30]. Fib and its related fragments are involved in tumor angiogenesis and metastasis [31]. Evidence has shown the association of elevated Fib levels with worse survival outcomes in gastrointestinal stromal tumors, pancreatic ductal adenocarcinoma, the coronavirus disease-19 (COVID-19), and colon cancer [32–35]. Similarly, in patients with UTUC, elevated plasma Fib levels are an independent predictor of poor survival [36]. In the present study, we found that Fib was a significant independent prognostic factor of UTUC following curative resection. Creatinine, the end product of creatine and creatine phosphate metabolism, is excreted mainly by the kidney [37–39]. Accumulating evidence indicates that oxidative stress plays a central role in the pathogenesis of chronic kidney disease [40]. In the case of renal dysfunction, the daily creatinine produced is not completely excreted, resulting in increased blood creatinine levels. Thus, values of CRE can reflect the oxidative stress injury of an organism. In this research, serum creatinine level was identified as an independent risk factor for mortality.

*γ*-GGT is a ubiquitous cell surface enzyme that plays a crucial role in antioxidant defense systems [41]. Based on epidemiological and experimental studies, elevated serum *γ*-GGT might be an early and sensitive marker for oxidative stress [42, 43]. *γ*-GGT levels have been shown to have independent prognostic value in various types of cancer, including non-small lung cancer, colorectal cancer, and hepatocellular carcinoma [44–46]. In line with previous studies, we found that a high serum *γ*-GGT level was an independent prognostic factor for OS in UTUC patients. LDH is a tetrameric enzyme comprising two major subunits, A and/or B. Lactate dehydrogenase A (LDHA) is a key enzyme in aerobic glycolysis that preferentially converts pyruvate to lactic acid [47]. Tumor cells overexpress LDHA to obtain energy through aerobic glycolysis [48]. Le et al. reported that a reduction in LDHA level or activity triggers oxidative stress and cell death [49]. In the current study, we showed that LDH is an effective indicator for predicting prognosis in UTUC patients. ALB is the most abundant plasma protein and has important antioxidant activities [50]. Our results were consistent with a previous report that ALB is an independent survival risk factor [51]. In summary SOSIs which consists of Fib, CRE, *γ*-GGT, ALB, and LDH, is a newly established scoring tool for representing the status of systematic oxidative stress. Our results demonstrate that high SOSIs correlates significantly with decreased OS and PFS in patients with UTUC.

Currently, the standard of care for patients with nonmetastatic MIBC is platinum-based neoadjuvant chemotherapy in combination with radical cystectomy [52]. However, there is currently no high-level evidence to support use of neoadjuvant chemotherapy for high-risk UTUC, given the significant bias and heterogeneity. A recent randomized controlled trial assessing the benefit of adjuvant gemcitabine–platinum combination chemotherapy initiated within 90 days after RNU reported significant improvement in disease-free survival for patients. In this study, we found no significant difference between oxidative stress and use of chemotherapy; chemotherapy did not improve survival. The possible explanation is that the number of people who received chemotherapy was small, and the treatment plan was not uniform due to the long-time span of this study. In addition, immune checkpoint inhibitors have been used in treatment of urothelial carcinoma to improve overall survival and reduce morbidity and mortality [53]. We hope to collect immunotherapy data for analysis in a future study.

There are some limitations to our study. First, as with all retrospective studies, the limitations of our study are inherent to the design. There was also a limited number of patients; thus a high risk of selection bias may exist in the findings. Randomized controlled trials with a larger population are warranted to validate the prognostic ability of SOSIs in patients with UTUC. Moreover, it was difficult to guarantee the consistency of the pathologic outcomes, because the patients in our study were treated by multiple surgeons. In this study, no information on the inflammatory status of patients was analyzed, which may affect the SOSIs level. In addition, all patients enrolled in this study were Chinese, and the influence of ethnic diversity cannot be ignored. The findings must be further explored in future studies. Finally, there is currently a lack of data on immunotherapy, and we could not compare levels of oxidative stress in patients with immune checkpoint inhibitors. Of course, we intend to continue this line of inquiry in a follow-up study.

## Conclusion

To our knowledge, this is the first study to explore the prognostic value of SOSIs for survival in patients with UTUC who underwent RNU. We confirmed that SOSIs can sever as an independent predictor of OS and PFS in patients with UTUC. The nomogram also showed good predictive performance in UTUC patients. We thus believe that use of the SOSIs may facilitate preintervention risk stratification in UTUC.

## Data Availability

All data generated or analyzed during this study are included in this published article.
