# Correction: Systematic oxidative stress indices predicts prognosis in patients with urothelial carcinoma of the upper urinary tract after radical nephroureterectomy

**DOI:** 10.1186/s40001-023-01582-w

**Published:** 2023-12-21

**Authors:** Jianyong Liu, Shicong Lai, Pengjie Wu, Jiawen Wang, Jianye Wang, Jianlong Wang, Yaoguang Zhang

**Affiliations:** 1 Department of Urology, Beijing Hospital, National Center of Gerontology, Institute of Geriatric Medicine, Chinese Academy of Medical Sciences, No. 1 DaHua Road, Dong Dan, Beijing, 100730 People’s Republic of China; 2 Graduate School of Peking Union Medical College and Chinese Academy of Medical Sciences, Beijing, People’s Republic of China; 3 Beijing Hospital Continence Center, Beijing, People’s Republic of China; 4 Department of Urology, Peking University People’s Hospital, Beijing, 100044 People’s Republic of China

**Correction: European Journal of Medical Research (2023) 28:469** 10.1186/s40001-023-01295-0

In the original publication of the article [1], the affiliation 1 was incorrectly published as “Department of Urology, Institute of the Geriatric Medicine, Beijing Hospital, National Center of Gerontology, Chinese Academy of Medical Sciences, No. 1 DaHua Road, Dong Dan, Beijing 100730, China”. The corrected affiliation 1 should read as “Department of Urology, Beijing Hospital, National Center of Gerontology, Institute of Geriatric Medicine, Chinese Academy of Medical Sciences, No. 1 DaHua Road, Dong Dan, Beijing 100730, People’s Republic of China”. The original article has been corrected.
